# Rosiglitazone use and associated adverse event rates in Canada between 2004 and 2010

**DOI:** 10.1186/1756-0500-6-82

**Published:** 2013-03-05

**Authors:** Nigel SB Rawson, Jorge A Ross Terres

**Affiliations:** 1 Medical Affairs, GlaxoSmithKline Inc., 7333 Mississauga Road, Mississauga, ON, L5N 6L4, Canada; 2 Eastlake Research Group, 511 Maple Grove Road, P.O. Box 61028, Oakville, ON, L6J 7P5, Canada

**Keywords:** Rosiglitazone, Prescription data, Adverse events, Risk communication

## Abstract

**Background:**

We examined the change in the use of rosiglitazone-containing products (RCPs) Canada-wide between 2004 and 2010 and whether the rates of adverse events in association with RCP therapy in Canadian patients changed in this period to better understand the real world use of RCP medications and as part of a regulatory commitment by GlaxoSmithKline to Health Canada to assess whether there was an impact of a risk communication on cardiac safety.

**Methods:**

RCP utilization data were obtained from IMS Brogan’s longitudinal de-identified patient database (known as LRx) that tracks prescription activity using store-based data collection from pharmacies in all Canadian provinces. Adverse events (AEs), serious adverse events (SAEs) and cardiac AEs associated with RCP use in Canadian patients between April 2004 and December 2010 were identified from GlaxoSmithKline’s AE database and, using the LRx data, rates per 100,000 patients were estimated.

**Results:**

A total of 239,184 patients were identified as having received at least one RCP prescription between 2004 and 2010 from the LRx. After excluding those with inconsistent gender or age, only one RCP prescription at the pharmacy, a prescription from a pharmacy that had not consistently reported for the past six years or an unreasonably high number of prescriptions, 180,936 patients remained for the analysis. The number of reports identified from the AE database that occurred between April 2004 and December 2010 was 1,037. The average monthly rates of AEs, SAEs and cardiac AEs decreased by 57%, 43% and 4%, respectively, between the observed periods, April 2004-October 2007 and November 2007-December 2010.

**Conclusions:**

The findings of this analysis demonstrate a significant decrease in RCP use in Canada following a meta-analysis publication suggesting harm, which has been maintained. It is not possible to disentangle whether the continuing decline can be attributed to the meta-analysis, the changes in prescribing guidelines, media attention or a combination of some or all of these factors.

## Background

Following the publication in May 2007 of a meta-analysis [1] that suggested an increased risk of myocardial infarction associated with rosiglitazone-containing products (RCPs), a dramatic decrease in the use of RCPs has been demonstrated in the United States and Europe [2-5]. In Canada, two reports from Ontario and British Columbia showed a similar decline in RCP use in those provinces [6,7]. However, neither examined whether there was an effect associated with a risk communication on cardiac safety issued by Health Canada in November 2007 [8], one component of which was that rosiglitazone “is no longer approved as monotherapy for type 2 diabetes.” There was also a reminder that RCPs are not indicated for use as a component of a triple therapy (i.e. in combination with metformin and a sulfonylurea) diabetes regimen.

To better understand the real world use of RCP medications and as part of a regulatory commitment by GlaxoSmithKline (GSK) to Health Canada to assess whether there was an impact of the risk communication across Canada in terms of decreasing use of RCPs as monotherapy or as part of triple or triple-plus therapy, we examined the change in the use of RCPs Canada-wide between 2004 and 2010. The commitment also required an evaluation of the rates of adverse events (AEs), especially cardiac AEs, in association with RCP therapy in Canadian patients before and after the release of the risk communication.

## Methods

The only cross-Canada resource for drug utilization commercially available is IMS Brogan’s longitudinal de-identified patient database (known as LRx), which tracks prescription activity using store-based data collection from pharmacies in all Canadian provinces [9]. LRx covers 63% of prescriptions nationally and is recognized as a critical national information source [10].

Patients dispensed prescriptions for RCPs, other oral anti-diabetics or insulin between 2004 and 2010 were identified from LRx and those dispensed RCPs alone or in combination retained by IMS Brogan. Since LRx only began in 2004 and data from the first three months are misleading because they demonstrate the building of the database rather than real utilization, the number of RCP patients per month was calculated from April 2004 onwards. Since it is the usual way that IMS Brogan provides data and because a national picture was required, proportional allocation procedures established by IMS were used to provide an estimate of overall national RCP use and as monotherapy, in combination with another oral anti-diabetic or insulin (dual therapy), or in combination with multiple other anti-diabetic products (triple-plus therapy) in the Canadian population of 34.5 million. These data were provided to GSK.

AEs, serious adverse events (SAEs) and cardiac AEs associated with RCP use in Canadian patients between April 2004 and December 2010 were identified from GSK’s AE database and, using the LRx data, rates per 100,000 patients were estimated. The AE database has been approved by and information from it is shared with regulatory agencies. It has been shown to be more comprehensive than both the US Food and Drug Administration and the World Health Organization adverse reaction databases for GSK’s products [11]. Nevertheless, since the data come from voluntary reporting, they are undoubtedly under-reported. In addition, the AE data did not specify each patient’s RCP treatment regimen.

Since the data used in this analysis were de-identified, ethics approval and patient consent were not required. Moreover, because the analysis was focused on the impact of the Health Canada risk communication on a change in prescribing requirements and cardiac AEs, information on fractures or other AEs was not collected.

## Results

A total of 239,184 patients were identified as having received at least one RCP prescription between 2004 and 2010 from the LRx. After excluding those with inconsistent gender or age (11,868), only one RCP prescription at the pharmacy (9,669), a prescription from a pharmacy that had not consistently reported for the past six years (35,347), or an unreasonably high number of prescriptions (1,364), 180,936 patients remained. These exclusions remove a significant number of patients, but we chose to be confident in the data that we used rather than unsure about the reliability of part of the data.

**Figure 1:**
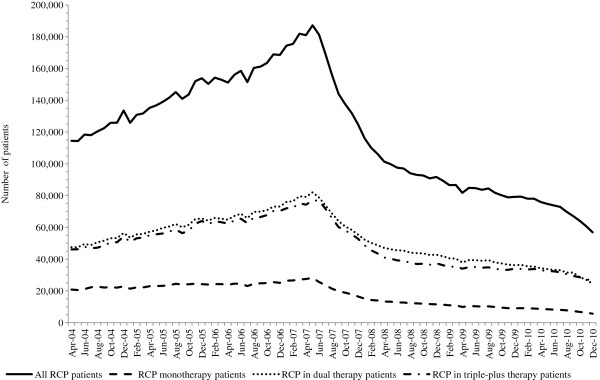
Number of patients receiving a rosiglitazone-containing product (RCP) by month, April 2004 to December 2010.

The monthly number of RCP patients increased by 63% between April 2004 and the peak in May 2007. The number subsequently decreased from the peak by 26% in October 2007, 41% in February 2008, 58% in December 2009 and 70% in December 2010 (Figure 1). The monthly numbers of mono, dual and triple-plus therapy patients are also shown in Figure 1. Between May 2007 and December 2010, mono, dual and triple-plus therapy decreased by 80%, 70% and 65%, respectively.

The number of reports identified from GSK’s AE database that occurred in Canadian patients between April 2004 and December 2010 was 1,037. Monthly rates of AEs, SAEs and cardiac AEs (per 100,000 patients) were estimated and the averages of these in two time periods (April 2004 to October 2007 and November 2007 to December 2010) calculated (Table 1). The average monthly rates of AEs, SAEs and cardiac AEs decreased by 57%, 43% and 4%, respectively, between the two periods, but there were large overlapping confidence intervals.

**Table 1:** Estimated average monthly rates of adverse events per 100,000 patients in two time periods

	**April 2004 - October 2007**			**November 2007 - December 2010**		
	**Average**	**Range**	**95% CI**	**Average**	**Range**	**95% CI**
Adverse events	13.4	2.3-27.3	8.6-20.8	5.7	0.0-12.5	2.4-13.6
Serious adverse events	6.0	0.6-21.5	3.1-11.5	3.4	0.0-8.7	1.1-10.6
Cardiac adverse events	2.3	0.0-9.4	0.8-6.5	2.2	0.0-6.6	0.5-8.9

## Discussion

The dispensing of RCPs across Canada decreased substantially from the peak in May 2007 to October 2007 and continued to decline after the November 2007 risk communication [8] through to December 2010. These results are consistent with other analyses in Canada [6,7] and with studies in the United States and Europe [2-5]. However, due to the limited information available, it was not possible to assess whether the risk communication had any additional impact on an already decreasing utilization pattern.

The largest decrease was in the monotherapy group and the smallest in the triple-plus therapy group. The former is consistent with the change in prescribing guidelines [8]. The latter is not, but it should be recognized that patients on triple-plus therapy are likely to be further along the diabetes disease progression pathway and to require multiple drugs to maintain glycemic control. In such patients, it may have been extremely difficult to find an effective product to replace the RCP. Access to data on the use of other anti-diabetic drugs may have provided greater insight into whether the risk communication impacted therapy.

There was no evidence of an increased rate of adverse events following the publication of the Health Canada risk communication, which might have been anticipated since patients potentially at risk may have been the ones who discontinued RCP therapy. In fact, there was an apparent decrease in the rate of all AEs and serious AEs. The rate of cardiac AEs was already low so that a similar decline was not likely to occur. Despite the comprehensiveness of the GSK AE database [11], the estimated rates of adverse events are impacted by small numbers, potentially due to under-reporting, and by the relatively crude but best available method used to calculate them, which is a significant limitation of this analysis. The lack of information on each patient’s RCP treatment regimen in the AE data was a further limitation.

## Conclusions

The findings of this analysis demonstrate a major decrease in RCP use in Canada following the meta-analysis publication, which has been maintained. It is not possible to disentangle whether the continuing decline can be attributed to the meta-analysis, the changes in prescribing guidelines, media attention or a combination of some or all of these factors.

## Competing interests

At the time of writing, the authors were both full-time employees of GlaxoSmithKline Inc.

## Authors’ contributions

Both authors contributed to the design of the study, identified and obtained the data. NSBR wrote the first draft of the manuscript and coordinated the production of the final manuscript. Both authors reviewed the results of the analyses and contributed to, read and approved the final manuscript.

## Authors’ information

NSBR was an epidemiologist and JART is Director, Specialty Care in Medical Affairs, GlaxoSmithKline Inc., Mississauga, Ontario, Canada. NSBR left GlaxoSmithKline Inc. in March 2012 and is now President, Eastlake Research Group, Oakville, Ontario, Canada.
